# Quantification of Potential Inequities in Breast Cancer Incidence in New Mexico Through Bayesian Disease Mapping

**DOI:** 10.5888/pcd18.200468

**Published:** 2021-03-11

**Authors:** David Zahrieh, Michael A. Golafshar, Samir H. Patel, Todd A. DeWees

**Affiliations:** 1 Department of Health Sciences Research, Mayo Clinic, Rochester, Minnesota; 2 Department of Health Sciences Research, Mayo Clinic, Scottsdale, Arizona; 3 Department of Radiation Oncology, Mayo Clinic, Phoenix, Arizona

## Abstract

**Introduction:**

The incidence of breast cancer among non-Hispanic American Indian and Alaska Native (AI/AN) women varies across the United States. We applied county-level Bayesian disease mapping to quantify potential inequities in 10-year breast cancer incidence in New Mexico to better inform health equity initiatives among its non-Hispanic at-risk AI/AN population.

**Methods:**

We used data from the Surveillance, Epidemiology, and End Results (SEER) program from 2005 through 2014 to identify new cases of breast cancer in New Mexico’s 33 counties. To account for spatial variation, a county-level Area Deprivation Index, and the small area estimation problem inherent in these data, we borrowed strength globally and locally by applying Bayesian disease mapping to the counts of age-adjusted county-level breast cancer incidence. We quantified the disparity effect, as measured by the age-adjusted rate ratio, comparing the incidence of breast cancer between at-risk non-Hispanic AI/AN and non-Hispanic White women and assessed whether the ratio differed among counties.

**Results:**

Accounting for over-dispersion and spatial correlation among the 33 counties and a county-level Area Deprivation Index, the posterior mean of the overall age-adjusted rate ratio was 0.384 (95% credible interval, 0.253­–0.546). The age-adjusted rate of breast cancer in non-Hispanic AI/AN women was 0.38 times the corresponding age-adjusted rate for non-Hispanic White women; however, a significant reduction in breast cancer incidence was observed in 16 of the 33 counties.

**Conclusion:**

The application of Bayesian disease mapping to these data provided substantial evidence of an overall disparity in breast cancer incidence between at-risk non-Hispanic AI/AN and non-Hispanic White women in New Mexico, which was more marked than previously reported and limited to certain counties. Targeted statewide and county-level health-equity initiatives may lead to a reduction in these disparities.

**Summary**

**What is already known on this topic?**

Breast cancer incidence among non-Hispanic American Indian and Alaska Native (AI/AN) women has been quantified in large geographic regions of the United States, showing substantial regional variation in incidence inequities among non-Hispanic AI/AN populations.

**What is added by this report?**

We found substantial evidence in New Mexico of an overall reduction in breast cancer incidence among at-risk non-Hispanic AI/AN women compared with non-Hispanic White women in certain counties in the state.

**What are the implications for public health practice?**

Our findings can facilitate targeted statewide and county-level cancer control interventions to mitigate such disparities.

## Introduction

In the past 2 decades, substantial progress has been made in the United States in reducing breast cancer death rates for non-Hispanic White women; however, this reduction has not been shared by non-Hispanic American Indian and Alaska Native (AI/AN) women (1). The corresponding mortality-to-incidence ratio is higher for non-Hispanic AI/AN women than for non-Hispanic White women (1). This inequity has persisted despite breast cancer being amenable to screening and treatment. A possible contributor to the higher mortality-to-incidence ratio may be a lower prevalence of mammography use among non-Hispanic AI/AN women compared with non-Hispanic White women (2). Mammography can detect breast cancer in its early stages when it may respond better to treatment (3). Although use of mammography has recently increased among non-Hispanic AI/AN women, its use remains below Healthy People 2020 targets and lower than among other racial/ethnic subgroups (4).

An important indicator of health status in the non-Hispanic AI/AN population is breast cancer incidence. This incidence has primarily been quantified in large geographic regions of the United States (1,5,6). The less favorable regional-level breast cancer incidence rates reported among non-Hispanic AI/AN versus non-Hispanic White women in the southwestern region of the United States underscore the need to continue to quantify potential inequities in breast cancer outcomes, and at a more granular county level, to facilitate targeted cancer control interventions to mitigate such disparities.

Our aim was to quantify potential disparities in breast cancer incidence between non-Hispanic AI/AN women and non-Hispanic White women in New Mexico overall and in each of its 33 counties during our 10-year study period, 2005 through 2014. New Mexico has 23 federally recognized tribes and, based on 2015 estimates, AI/ANs make up nearly 10.5% of the state’s population (7). Because we were interested in obtaining precise local estimates of breast cancer incidence among each racial/ethnic group at the county level as well as assessing broad trends across the state, we used Bayesian disease mapping, which can be implemented to account for spatial variation. It can also account for county-level covariates when quantifying such potential inequities and can address the small area estimation problem inherent in these data by borrowing strength globally and locally across the state (8).

By using Surveillance, Epidemiology, and End Results (SEER) (9) program data from 2005 through 2014, we applied Bayesian disease mapping to address 3 study-specific questions to quantify potential inequities in 10-year breast cancer incidence in New Mexico:

1. Is the overall incidence of breast cancer among at-risk non-Hispanic AI/AN women excessively low compared with non-Hispanic White women?
2. Does the rate ratio, comparing the incidence of breast cancer between at-risk non-Hispanic AI/AN women and non-Hispanic White women, differ among New Mexico counties?
3. Do some counties in New Mexico have a lower breast cancer incidence among at-risk AI/AN women than would be expected?

Research into these questions can contribute to planning public health services and interventions in New Mexico that may lead to reducing disparities in breast cancer outcomes among non-Hispanic AI/AN at-risk women.

## Methods

### Data preparation

**Figure 1:**
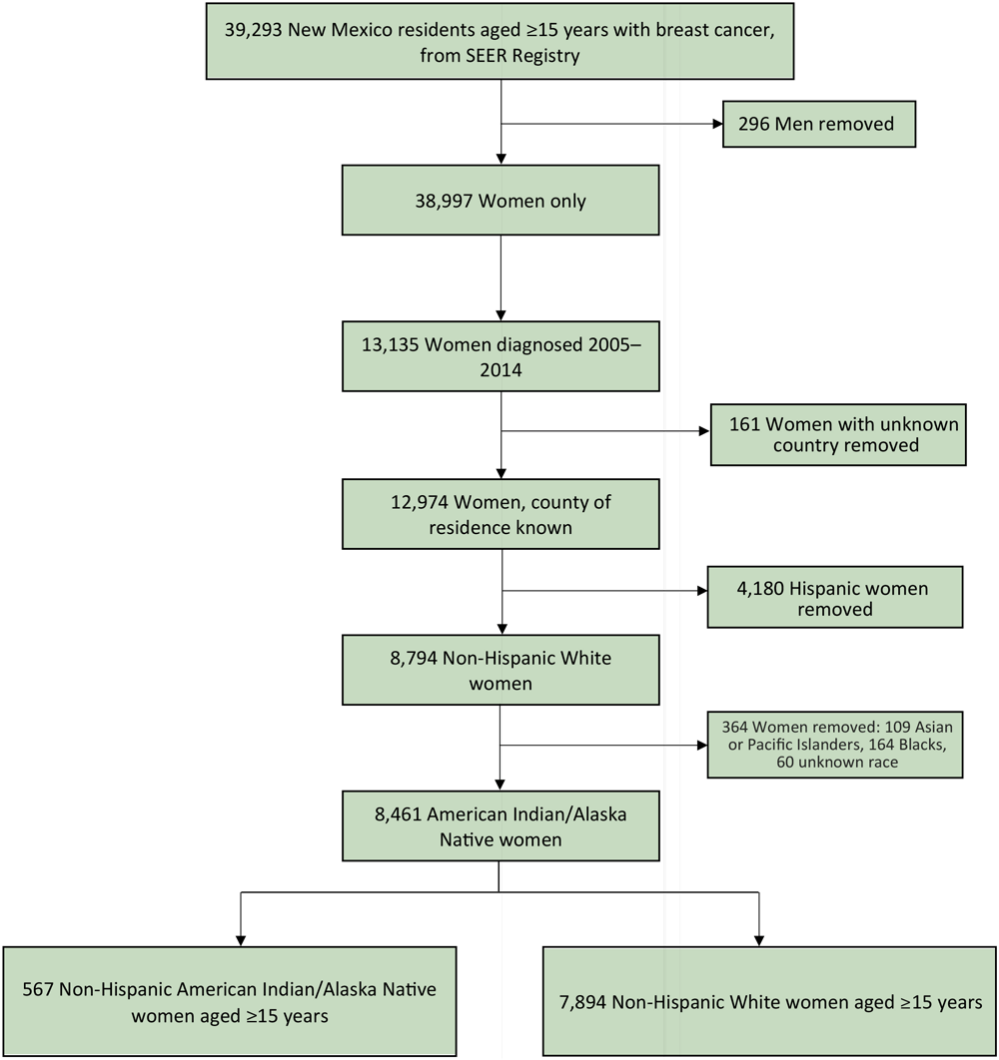
Data flow diagram describing the selection of New Mexico women with breast cancer for inclusion in a study of potential inequities in breast cancer incidence among non-Hispanic American Indian/Alaska Native and non-Hispanic White women. Abbreviation: SEER, Surveillance, Epidemiology, and End Results program.

Data were limited to AI/AN and White women of non-Hispanic origin who were aged 15 years or older (Figure 1). From the data on the 38,997 women in the SEER registry who received a diagnosis of breast cancer, 13,135 were diagnosed from 2005 through 2014. County of residence was known for 12,974 of these women, of whom 8,794 were of non-Hispanic origin. After excluding other racial/ethnic groups (109 Asian or Pacific Islander, 164 Black, and 60 unknown race), our population surveillance data consisted of 8,461 women with breast cancer (567 non-Hispanic AI/AN women, 7,894 non-Hispanic White women) diagnosed from 2005 through 2014 across the 33 counties. Data on the number of women at risk were obtained from US Census Bureau data for 2010, the midpoint of our study period, and retrieved in 5-year age intervals (eg, 15–19 y). Although the risk of breast cancer for women aged 15 to 17 is low, to avoid excluding at-risk women aged 18 and 19, we retrieved the number at risk in the 15 to 19 age interval in addition to all higher 5-year age intervals. Therefore, we considered 443,814 non-Hispanic AI/AN and non-Hispanic White women aged 15 years or older at risk for breast cancer. These data were extended to include county-level Area Deprivation Index (ADI) scores developed by Mayo Clinic researchers (10), which were measured from 17 indicators that served as a surrogate for income, employment, housing, and education.

### Statistical analysis

To address questions on disparate incidence, we applied a single Bayesian Poisson hierarchical model to model the county-level, age-adjusted number of women with breast cancer defined as

$$y_{ir}^{*}\;=\;\sum_{a}\pi_{a}y_{iar}$$

where *y_iar_* denotes the number of women with breast cancer in county *i*, age group *a*, racial group *r*, and *π_a_* denotes the proportion of the 2010 US female population aged 15 years or older in age group *a*. Here, *i *ranges from 1 to 33; *a ∈ {15 − 19, 20 − 24, …, 80 – 84, 85*+} and *r = 0* (non-Hispanic White population) or *r = 1* (non-Hispanic AI/AN population). Also, let *n_ir_* denote the population size at risk in the *i^th^* county for the *r^th^* racial group. Then, the hierarchical model proposed to address our study-specific questions can be expressed as

$$y_{ir}^{*}\sim \;Poisson\left(n_{ir}\lambda_{ir}\right),\;i\;=\;1,\;\ldots ,\;33;r\;=\;0,\;1$$

log (*λ_ir_*) = *β_0_*
+ *β_1_x_1_*
+ *β_2_x_2_*
+ *β_3_x_1_x_2_* + *θ_ir_* + *ϕ_ir_*

with *x_1_* denoting an indicator variable, which takes the value 1 if *r* = 1 (non-Hispanic AI/AN population) and zero if *r* = 0 (non-Hispanic White population), and *x_2_* denoting an indicator variable, which takes the value 1 if county-level ADI is in the lower 20th percentile and zero otherwise.

The inclusion of the interaction term permitted the disparity effect to be different at each level of the county-level variable *x_2_*_._ The prior distribution for each parameter *β_1_, β_2_, *and* β_3_* was set to a normal distribution with mean 0 and variance 1,000, while a flat prior was assumed for *β_0_*. The *θ_ir_* captured region-wide heterogeneity via an ordinary, exchangeable normal prior,


*θ_ir_ ~ N(0,τ_h_)*,

where *τ_h_* is a variance term. These random effects captured extra-Poisson variability (or over-dispersion) in the log-relative risks that varied globally (ie, over the entire state). Finally, the random effects *ϕ_ir_* are the parameters that make this a spatial model by capturing regional clustering. That is, they modeled extra-Poisson variability in the log-relative risks that varied locally, so that nearby counties would have more similar rates. Spatial association was defined through a neighborhood structure where 1 county was related to other counties that shared a common border and determined by an adjacency matrix. To specify the spatial association, we assumed a race-specific improper conditional autoregressive (CAR) (11) specification for *ϕ._r_* such that for each racial group *r* we have


*ϕ._r = _*_0_
*
~ *CAR(τ*_b_*;* r* = 0) and *ϕ._r = 1_ ~ CAR(τ_c_; r = 1*)

The variance parameters *τ_h_*, *τ_b_*, and *τ_c_* ****were given standard deviation uniform prior distributions in the range of 0 to 100 (12). The inclusion of both spatially uncorrelated (*θ*) and spatially correlated (*ϕ*) heterogeneity effects also addressed the small area estimation problem by borrowing strength globally and locally, respectively.

We used Markov chain Monte Carlo (MCMC) sampling to estimate posterior quantiles for the proposed Bayesian Poisson hierarchical model; R (R Foundation) and OpenBUGS (13) were used to fit the proposed hierarchical model. Estimated posterior quantiles were based on 3 chains, including a burn-in period for each chain. A long run of the sampler was required because of high levels of autocorrelation; therefore, samples were thinned by using only every fiftieth step in the sampler as a strategy for dealing with the otherwise overwhelming amounts of MCMC output (14). Consequently, posterior distributions were based on 60,000 samples, or 20,000 per chain. The deviance information criterion (DIC) was used to assess model adequacy (15). In our application, a difference in DIC greater than 2 was used to ascertain if the DIC was exhibiting a preference.

To answer our first question, we reported and interpreted the estimated posterior mean and 95% credible interval for the overall rate ratio defined as the ratio of the statewide average rates in each racial category (non-Hispanic AI/AN vs non-Hispanic White), or $\frac{{\overset{-}{\lambda }}_{\cdot 1}}{{\overset{-}{\lambda }}_{\cdot 0}}$, where ${\overset{-}{\lambda }}_{\cdot 1}\;=\;\frac{\sum_{i}n_{i1}\lambda_{i1}}{\sum_{i}n_{i1}}$ and ${\overset{-}{\lambda }}_{\cdot 0}\;=\;\frac{\sum_{i}n_{i0}\lambda_{i0}}{\sum_{i}n_{i0}}$. To answer the second question, we present the estimated posterior means and corresponding 95% credible intervals for each county-level rate ratio (non-Hispanic AI/AN vs non-Hispanic White women), defined as $\frac{\lambda_{i1}}{\lambda_{i0}}$ for the *i^th^* county. Finally, within the non-Hispanic AI/AN population, we reported and interpreted the estimated posterior mean and 95% credible interval for each county-level rate ratio (ie, the non-Hispanic AI/AN county-level rate vs the statewide average rate within non-Hispanic AI/AN), or $\frac{\lambda_{i1}}{{\overset{-}{\lambda }}_{\cdot 1}}$, to answer our last question.

## Results

**Figure 2:**
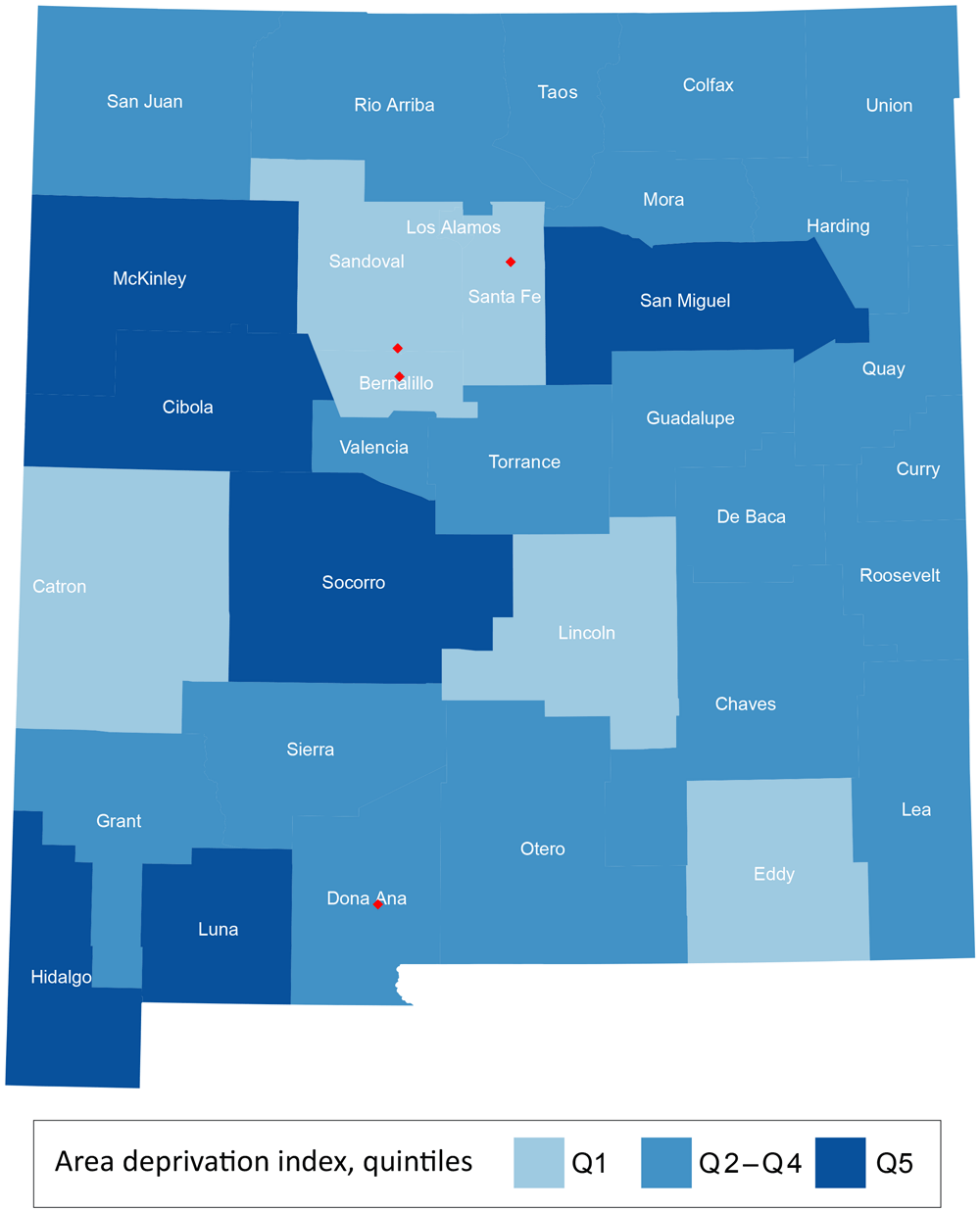
County-level Area Deprivation Index (ADI) quintiles for 33 New Mexico counties in 2012 categorized as the lower 20% (Q1), middle 60% (Q 2–4), and upper 20% (Q5). Higher quintiles indicate increased socioeconomic disadvantage. Red diamonds depict major cites (Albuquerque in Bernalillo County, Las Cruces in Dona Ana County, Rio Rancho in Sandoval County, and Santa Fe in Santa Fe County). ADI scores were developed by Mayo Clinic researchers (10), and are derived from 17 indicators that served as surrogates for income, employment, housing, and education.

Of the 443,814 at-risk women in our 2010 sample, 75,048 (16.9%) were non-Hispanic AI/AN. For each of the 33 New Mexico counties in New Mexico, we calculated the county-level ADI based on 2012 socioeconomic data according to the lower 20%, middle 60%, and upper 20% (Figure 2). The 20th and 80th percentile ADI were 101.6 and 120.8, respectively, and the median ADI was 110.2 (range, 39.2–149.8); higher values correspond to increased socioeconomic disadvantage.

**Table:** 

County	Rate Ratio, Non-Hispanic AI/AN vs Non-Hispanic White Women
	Posterior Mean Rate Ratio (95% Credible Interval Rate Ratio)
Bernalillo	0.311 (0.121–0.600)
Catron	0.346 (0.066–0.927)
Chaves	0.446 (0.092–0.991)
Cibola	0.440 (0.175–0.834)
Colfax	0.488 (0.109–1.284)
Curry	0.490 (0.064–1.348)
De Baca	0.460 (0.095–1.073)
Dona Ana	0.407 (0.066–0.910)
Eddy	0.372 (0.058–1.061)
Grant	0.432 (0.067–1.062)
Guadalupe	0.456 (0.108–1.037)
Harding	0.477 (0.112–1.162)
Hidalgo	0.492 (0.048–1.436)
Lea	0.480 (0.069–1.215)
Lincoln	0.335 (0.081–0.822)
Los Alamos	0.370 (0.080–1.072)
Luna	0.432 (0.063–1.058)
McKinley	0.462 (0.228–0.808)
Mora	0.460 (0.132–1.044)
Otero	0.437 (0.112–0.930)
Quay	0.455 (0.109–1.026)
Rio Arriba	0.416 (0.131–0.816)
Roosevelt	0.463 (0.085–1.087)
San Juan	0.478 (0.250–0.820)
San Miguel	0.465 (0.132–1.078)
Sandoval	0.339 (0.120–0.715)
Santa Fe	0.326 (0.106–0.737)
Sierra	0.419 (0.092–0.903)
Socorro	0.422 (0.106–0.860)
Taos	0.492 (0.139–1.296)
Torrance	0.432 (0.123–0.889)
Union	0.499 (0.092–1.341)
Valencia	0.421 (0.100–0.882)

**Figure 3:**
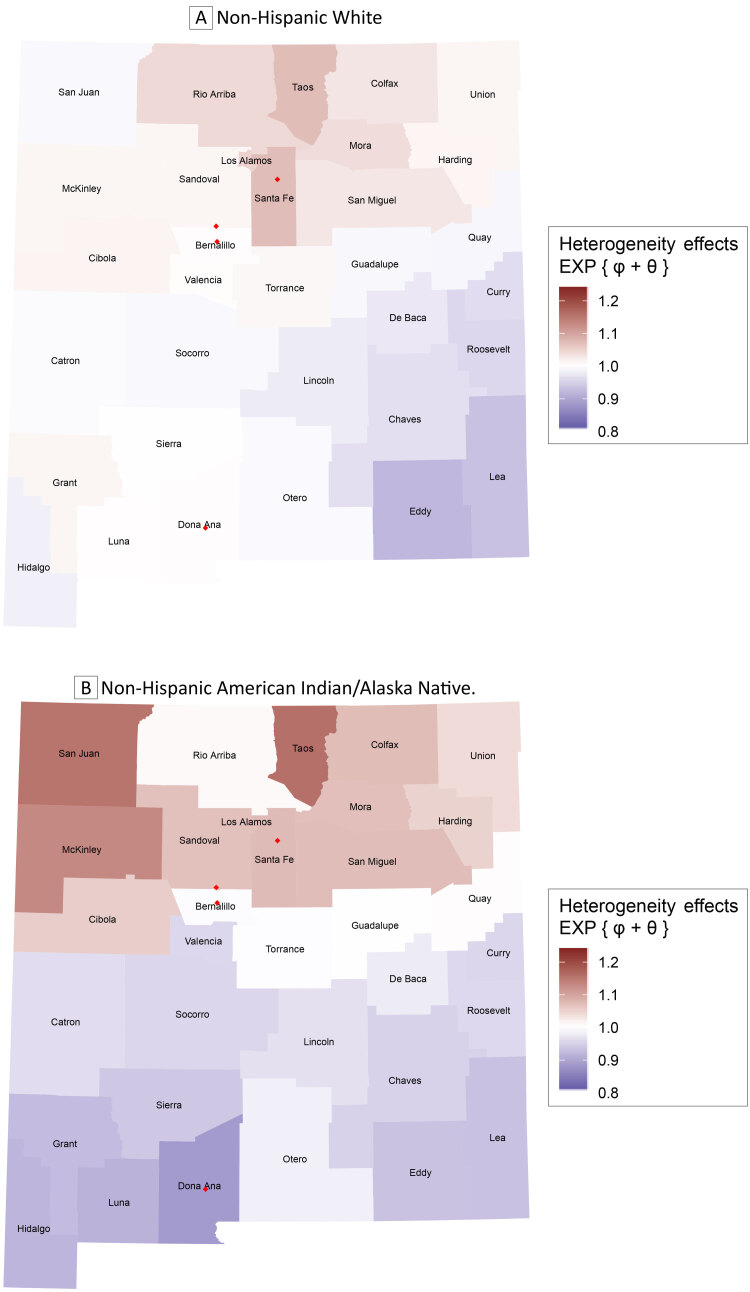
Exponentiated sum of the posterior average county-level heterogeneity effects obtained from the proposed model. Map A gives the results for non-Hispanic White women and Map B gives the results for non-Hispanic American Indian/Alaska Native women. Red diamonds depict major cites (Albuquerque in Bernalillo County, Las Cruces in Dona Ana County, Rio Rancho in Sandoval County, and Santa Fe in Santa Fe County).

In the estimated posterior quantities from fitting the proposed model (Table 1), the expected [standard deviation] estimate of the standard deviation associated with the spatially correlated heterogeneity effects suggested strong spatial patterning in the non-Hispanic AI/AN population (*√τ_c_*: 0.567 [0.499]), whereas this was not the case in the non-Hispanic White population (*√τ_b_*: 0.143 [0.110]). We mapped the sum of the posterior averages of the county-specific random effects *ϕ* and *θ* that were exponentiated within the non-Hispanic White population and the corresponding non-Hispanic AI/AN population (Figure 3). Interpreting the excess variability observed in the data in this fashion isolates the upper half of the state as an area of generally increased risk of breast cancer diagnosis in both maps, but those northern areas of increased risk are largely concentrated in the north-central regions in the non-Hispanic White population; furthermore, the northern areas of elevated risk of breast cancer diagnosis are more pronounced in the non-Hispanic AI/AN population. Although the areas of low risk of breast cancer diagnosis in the non-Hispanic AI/AN population are seen across the lower half of the state, the regions of low risk within the non-Hispanic White population are confined to the southeastern portion of the state.

**Table 1:** Estimated Posterior Quantities From Fitting the Proposed Model, New Mexico Breast Cancer Incidence Study, 2005–2014^a^

Description of Explanatory Variable	Parameter	Mean (SD)	95% Credible Interval
**Intercept**	*β_0_*	−7.009 (0.090)	(−7.189 to −6.836)
*x_1_* non-Hispanic AI/AN vs non-Hispanic White	*β_1_*	−0.937 (0.360)	(−1.763 to −0.346)
*x_2_* ADI in lower 20th percentile vs otherwise	*β_2_*	0.229 (0.132)	(−0.035 to 0.486)
2-Way interaction (*x_1_* × *x_2_*)	*β_3_*	−0.303 (0.551)	(−1.373 to 0.812)
**Random effects**			
Spatial component: non-Hispanic White	*√τ_b_*	0.143 (0.110)	(0.003 to 0.411)
Spatial component: non-Hispanic AI/AN	*√τ_c_*	0.567 (0.499)	(0.029 to 1.859)
Dispersion parameter	*√τ_h_*	0.070 (0.054)	(0.004 to 0.205)
Overall rate ratio (non-Hispanic AI/AN vs non-Hispanic White), ${\overset{-}{\lambda }}_{\cdot 1}/{\overset{-}{\lambda }}_{\cdot 0}$		0.384 (0.075)	(0.253 to 0.546)

Abbreviations: ADI, Area Deprivation Index; AI/AN, American Indian/Alaska Native.

a Estimates of posterior quantities were obtained from Markov chain Monte Carlo sampling. The 20th percentile for the ADI was 101.6; higher values correspond to increased socioeconomic disadvantage. The overall rate ratio was defined as the ratio of the average rates within each racial category (non-Hispanic AI/AN vs non-Hispanic white), or $\frac{{\overset{-}{\lambda }}_{\cdot 1}}{{\overset{-}{\lambda }}_{\cdot 0}}$, where ${\overset{-}{\lambda }}_{\cdot 1}\;=\;\frac{\sum_{i}n_{i1}\lambda_{i1}}{\sum_{i}n_{i1}}$ and ${\overset{-}{\lambda }}_{\cdot 0}\;=\;\frac{\sum_{i}n_{i0}\lambda_{i0}}{\sum_{i}n_{i0}}$ and *i *= 1, …, 33 corresponding to the 33 counties in New Mexico. The deviance information criterion was 160.9.

**Table:** 

County	Exponentiated Sum of the Posterior Average County-Level Heterogeneity Effects, EXP(*Φ* + *Θ*), Obtained From the Proposed Model	
	Non-Hispanic White Women	Non-Hispanic American Indian/Alaska Native Women
Bernalillo	1.003	0.996
Catron	0.995	0.960
Chaves	0.963	0.947
Cibola	1.012	1.055
Colfax	1.029	1.074
Curry	0.960	0.952
De Baca	0.972	0.976
Dona Ana	1.002	0.880
Eddy	0.914	0.928
Grant	1.010	0.921
Guadalupe	0.991	0.999
Harding	1.011	1.050
Hidalgo	0.983	0.913
Lea	0.928	0.927
Lincoln	0.977	0.964
Los Alamos	1.050	1.069
Luna	1.001	0.909
McKinley	1.010	1.133
Mora	1.039	1.071
Otero	0.993	0.981
Quay	0.990	1.002
Rio Arriba	1.042	1.008
Roosevelt	0.954	0.955
San Juan	0.992	1.158
San Miguel	1.027	1.073
Sandoval	1.010	1.070
Santa Fe	1.074	1.075
Sierra	1.001	0.935
Socorro	0.991	0.952
Taos	1.073	1.162
Torrance	1.009	0.997
Union	1.010	1.040
Valencia	1.003	0.953

The posterior mean of the overall, age-adjusted rate ratio was 0.384 (95% credible interval, 0.253–0.546). These data provide evidence of a significant overall disparity effect across New Mexico. The estimated rate of breast cancer in non-Hispanic AI/AN women was approximately 0.38 times the corresponding age-adjusted rate for non-Hispanic White women. Making allowance for the unobserved confounders *θ* and *ϕ* as well as allowing the disparity effect to be different across the 2 levels of the county-level ADI variable, we calculated the posterior mean for the age-adjusted rate ratio for each county on the basis of the proposed model (Table 2). For 16 of the 33 counties, the 95% credible intervals for the age-adjusted rate ratios were less than 1, while for the remaining 17 counties the 95% credible intervals included the null value of 1. It is worth noting that the 7 smallest posterior means for the age-adjusted rate ratios coincided with the 7 counties in the lowest 20th percentile for ADI. We also estimated posterior mean and 95% credible interval for each county-level rate ratio in the non-Hispanic AI/AN population. Compared with the average rate of breast cancer incidence in the non-Hispanic AI/AN population, the relative risk of breast cancer was largely constant across all counties, and all 95% credible intervals for the age-adjusted rate ratios were wide and included 1. Because the results depended on prior specifications, we examined sensitivity to prior specification. On the basis of these sensitivity analyses, our conclusions remained unchanged.

**Table 2:** County-Specific Posterior Means and 95% Credible Intervals for the Rate Ratios, New Mexico Breast Cancer Incidence Study, 2005–2014^a^

County	ADI Quintiles^b^	Non-Hispanic AI/AN vs Non-Hispanic White Women, Posterior Mean Rate Ratio (95% Credible Interval)	Non-Hispanic AI/AN Population, Posterior Mean Rate Ratio (95% Credible Interval)
Bernalillo	Q1	0.311 (0.121–0.600)	0.883 (0.387–1.527)
Catron	Q1	0.346 (0.066–0.927)	0.964 (0.204–2.428)
Chaves	Q2–Q4	0.446 (0.092–0.991)	0.963 (0.211–1.968)
Cibola	Q5	0.440 (0.175–0.834)	0.994 (0.459–1.637)
Colfax	Q2–Q4	0.488 (0.109–1.284)	1.121 (0.278–2.785)
Curry	Q2–Q4	0.490 (0.064–1.348)	1.051 (0.149–2.772)
De Baca	Q2–Q4	0.460 (0.095–1.073)	0.998 (0.228–2.123)
Dona Ana	Q2–Q4	0.407 (0.066–0.910)	0.915 (0.160–1.889)
Eddy	Q1	0.372 (0.058–1.061)	0.954 (0.160–2.556)
Grant	Q2–Q4	0.432 (0.067–1.062)	0.975 (0.162–2.225)
Guadalupe	Q2–Q4	0.456 (0.108–1.037)	1.009 (0.261–2.094)
Harding	Q2–Q4	0.477 (0.112–1.162)	1.075 (0.282–2.455)
Hidalgo	Q5	0.492 (0.048–1.436)	1.075 (0.114–2.979)
Lea	Q2–Q4	0.480 (0.069–1.215)	0.994 (0.153–2.346)
Lincoln	Q1	0.335 (0.081–0.822)	0.920 (0.242–2.049)
Los Alamos	Q1	0.370 (0.080–1.072)	1.093 (0.264–3.024)
Luna	Q5	0.432 (0.063–1.058)	0.967 (0.154–2.196)
McKinley	Q5	0.462 (0.228–0.808)	1.041 (0.662–1.471)
Mora	Q2–Q4	0.460 (0.132–1.044)	1.066 (0.346–2.254)
Otero	Q2–Q4	0.437 (0.112–0.930)	0.973 (0.273–1.915)
Quay	Q2–Q4	0.455 (0.109–1.026)	1.008 (0.264–2.093)
Rio Arriba	Q2–Q4	0.416 (0.131–0.816)	0.969 (0.341–1.677)
Roosevelt	Q2–Q4	0.463 (0.085–1.087)	0.986 (0.198–2.141)
San Juan	Q2–Q4	0.478 (0.250–0.820)	1.063 (0.696–1.519)
San Miguel	Q5	0.465 (0.132–1.078)	1.068 (0.338–2.290)
Sandoval	Q1	0.339 (0.120–0.715)	0.965 (0.387–1.849)
Santa Fe	Q1	0.326 (0.106–0.737)	0.987 (0.363–2.071)
Sierra	Q2–Q4	0.419 (0.092–0.903)	0.940 (0.222–1.825)
Socorro	Q5	0.422 (0.106–0.860)	0.936 (0.260–1.680)
Taos	Q2–Q4	0.492 (0.139–1.296)	1.180 (0.378–2.964)
Torrance	Q2–Q4	0.432 (0.123–0.889)	0.975 (0.305–1.812)
Union	Q2–Q4	0.499 (0.092–1.341)	1.124 (0.225–2.876)
Valencia	Q2–Q4	0.421 (0.100–0.882)	0.946 (0.247–1.772)

Abbreviation: ADI, area deprivation index; AI/AN, American Indian/Alaska Native; Q, quintile.

a The overall rate ratio (95% credible interval) comparing non-Hispanic AI/AN vs non-Hispanic white was 0.384 (0.253–0.546).

b The ADI for 2012; higher values correspond to increased socioeconomic disadvantage.

The DIC for the proposed model was 160.9. To assess the value of including the indictor variable for ADI in the proposed model, 2 additional models were considered, namely, the proposed model excluding the 2-way interaction term between the indicator variables for ADI and racial group (DIC = 158.1) and the proposed model excluding both the 2-way interaction term and the indicator variable for ADI (DIC = 162.8). The DIC expressed a preference for the reduced model that included the indicator variable for ADI. After adjusting for the unobserved confounders *θ* and *ϕ* and racial group, the posterior mean of the age-adjusted risk ratio, comparing the estimated rate of breast cancer between the ADI in the lower 20th percentile versus above, was 1.240 (95% credible interval, 0.952–1.578). These data suggest that the estimated rate of breast cancer in less socioeconomically disadvantaged counties, as defined by the lower 20th percentile, was 1.24 times the corresponding age-adjusted rate for counties more socioeconomically disadvantaged, although the 95% credible interval was not entirely to the right of the null value of 1. Based on this reduced model, the posterior mean of the overall age-adjusted rate ratio (non-Hispanic AI/AN vs non-Hispanic White women) was 0.385 (95% credible interval, 0.253–0.545), which was virtually the same as the overall age-adjusted rate ratio obtained from the proposed model that included the 2-way interaction term. Although the DIC expressed a preference for the model without the 2-way interaction term, we focused on the less parsimonious model that included the 2-way interaction term when quantifying the county-level age-adjusted rate ratios to permit a potential disparity effect to vary across the 2 levels of the county-level variable ADI.

## Discussion

We focused on quantifying potential inequities in 10-year breast cancer incidence in New Mexico and in each county to better inform health equity initiatives for non-Hispanic AI/AN women at risk for breast cancer. We used the age-adjusted rate ratio, comparing the incidence of breast cancer between at-risk non-Hispanic AI/AN and non-Hispanic White women, to quantify the disparity effect and based it on county-level age-adjusted counts of observed breast cancer cases diagnosed from 2005 through 2014 in New Mexico. Although traditional methods that calculate age-adjusted standardized incidence ratios are appropriate for large geographic areas, they are often unsuitable when the goal is to quantify local risk in small geographic areas, such as counties, while adjusting for potentially relevant covariate information; the local sample sizes in each county were too small to obtain reliable estimates with the desired levels of statistical precision by using traditional methods (16). To obtain a reliable estimate of the disparity effect in each county and overall, we applied Bayesian disease mapping to these population surveillance data. Bayesian disease mapping is a model-based approach that offered a means to improve county-level incidence estimates by borrowing more information from neighboring counties than from counties farther away, thereby smoothing extreme rates based on small local sample sizes toward local, neighboring values. Furthermore, this modeling-based approach accounted for the number of women at risk as well as a county-level ADI.

We found evidence of a substantial overall disparity effect across New Mexico. The age-adjusted rate of breast cancer among non-Hispanic AI/AN women was approximately 0.38 times the corresponding age-adjusted rate for non-Hispanic White women. The lower and upper limits of the corresponding 95% credible interval were 0.253 and 0.547, respectively. This overall finding appears in keeping with previous studies (1,5). By using age-adjusted breast cancer incidence rates in the southwest region for 2010 through 2015, Melkonian and colleagues in 2019 reported a corresponding age-adjusted rate ratio of 0.57 (5). Before that, White and colleagues in 2014 reported an age-adjusted rate ratio in the Southwest region of 0.49 during their study period, 1999–2009 (1).

Although previous studies have shown substantially lower breast cancer incidence rates among non-Hispanic AI/AN than non-Hispanic White women, there were regional differences in the age-adjusted rate ratios (1,5,17). In these epidemiologic studies, aggregated data over large geographic regions of the United States were used to quantify the age-adjusted rate ratios of breast cancer in each region. The southwest region comprises 5 states: Arizona, Colorado, Utah, Nevada, and New Mexico. Because activities such as health education, health statistics, and public health services are commonly implemented at the state rather than the regional level, we selected New Mexico for our study. Our findings can be directly accessible to New Mexico state health authorities to evaluate such disparities in their state and act to address them. Furthermore, federal funding for public health infrastructure such as mammography centers is commonly awarded at the state level so that knowing the state-level breast cancer burden in the non-Hispanic AI/AN population could facilitate targeted requests for federal and state funding. In our study, we observed a significant reduction in the age-adjusted breast cancer incidence rate in 16 of 33 New Mexico counties; 17 counties had no significant reduction.

Our study had limitations. First, although we were able to include county-specific covariates in our analytic approach, excess variability remained despite including our county-level ADI variable. We had considered 2 additional county-level covariates, an indicator for a health professional shortage area (whole area shortage vs none or partial) and an indicator for percentage of at-risk non-Hispanic AI/AN women (>5% vs ≤5%); however, adding these 2 county-level covariates to explain some of the spatial patterns in the county-level age-adjusted counts of breast cancer had a negligible effect. Identifying county-level covariates that have higher explanatory power has the potential to guide future measures to reduce disparities in breast cancer incidence. Second, although we wanted to highlight local, county-level detail and also capture broad trends across New Mexico, the county-level age-adjusted counts of breast cancer cases were sparse, leading to wide credible intervals, particularly when quantifying the county-level rate ratio in the non-Hispanic AI/AN population (ie, the non-Hispanic AI/AN county-level rate vs the statewide average rate within non-Hispanic AI/AN). Longer observation periods would likely mitigate this issue; furthermore, extending our model to account for temporal effects that may arise as a result of applying a longer observation period would be straightforward. A third limitation of our study is that our data were based on a 10-year time period 2005–2014, which means these data were 6 years old at the time this article was written. Socioeconomic, cultural, and health-system barriers to mammography among AI/AN women have been identified over the last decade (18–20), and interventions are in development to reduce such barriers and increase satisfaction among AI/AN women with mammography (21). Furthermore, access to breast cancer screening for non-Hispanic AI/AN women and medically underserved populations in general has increased through outreach strategies such as mobile mammography and the use of lay health advisors (22–24). More recent data may show that the gap in breast cancer incidence between non-Hispanic AI/AN and non-Hispanic White women has since been reduced.

Our application of Bayesian disease mapping to these population surveillance data from New Mexico provided substantial evidence of a significant overall reduction in the breast cancer incidence rate in at-risk non-Hispanic AI/AN women compared with non-Hispanic White women, which was more marked than previous reports. Targeted statewide health equity initiatives may reduce disparities in breast cancer incidence among non-Hispanic AI/AN women at risk for breast cancer, whereas targeted county-level initiatives may directly reduce disparities in breast cancer incidence.
